# Functional Nanomaterials and Nanocomposites for High-Performance Printed Biosensors

**DOI:** 10.3390/s26092646

**Published:** 2026-04-24

**Authors:** Minwoo Kim, Jeongho Shin, Seeun Yoon, Yongwoo Jang

**Affiliations:** 1 Department of Medical and Digital Engineering, College of Engineering, Hanyang University, Seoul 04763, Republic of Korea; kmw5478@hanyang.ac.kr; 2 Department of Biomedical Engineering, College of Engineering, Hanyang University, Seoul 04763, Republic of Korea; jjshin88@hanyang.ac.kr (J.S.); yse061214@hanyang.ac.kr (S.Y.); 3 Department of Pharmacology, College of Medicine, Hanyang University, Seoul 04763, Republic of Korea

**Keywords:** printed biosensors, nanomaterial inks, flexible electronics, electrochemical sensors, wearable sensors

## Abstract

Printed biosensors have attracted increasing attention as platforms for rapid, low-cost, and portable diagnostics because they can be fabricated on flexible or rigid substrates using scalable printing techniques. Their performance is strongly influenced by both the printing process and the materials employed, since factors such as ink rheology, particle dispersion, interfacial behavior, and post-processing conditions directly affect device architecture, sensing performance, and manufacturing reliability. This review summarizes recent advances in printed biosensors from the combined perspectives of printing technologies and functional materials. Commonly employed printing techniques, including inkjet, screen, aerosol jet, and roll-to-roll gravure printing, are discussed with emphasis on their processing characteristics and material requirements. The review also examines key material platforms used in printed biosensors, including carbon-based nanomaterials, metal oxides, metal nanoparticles, conductive polymers, dielectric materials, and hybrid composites, highlighting their roles in electrical conductivity, catalytic activity, biomolecule immobilization, mechanical flexibility, and overall analytical performance. Finally, current challenges and emerging research directions are outlined with respect to ink stability, post-processing strategies, sensor reliability, manufacturability, and practical translation. Overall, this review emphasizes that the development of high-performance printed biosensors depends on the synergistic integration of rational material design with optimized printing strategies.

## 1. Introduction

Recently, printed biosensors have attracted considerable attention as promising platforms for rapid, low-cost, and portable diagnostics [1,2,3]. Unlike conventional biosensors, which are fabricated using complex microfabrication processes, printed biosensors can be produced by depositing functional materials directly onto flexible or rigid substrates using scalable printing techniques [4,5,6]. This approach offers several practical advantages, including reduced material consumption, simple patterning, compatibility with large-area manufacturing, and suitability for disposable point-of-care devices [7,8,9]. At the core of these advances lies the development of functional materials. In printed biosensors, material selection plays a critical role in determining device performance, printability, and practical applicability [10,11]. The conductivity of the electrode, catalytic activity toward target analytes, surface chemistry for biomolecule immobilization, mechanical flexibility of the sensing layer, and long-term operational stability are governed by the properties of the materials used [12,13]. Therefore, a wide range of material platforms—including carbon nanomaterials, metal oxides, metal nanoparticles, conductive polymers, dielectric materials, and hybrid composites—have been extensively investigated to meet these functional requirements [14,15,16,17]. In addition, each printing method imposes distinct constraints on ink rheology, particle size, solvent system, interfacial behavior, and post-processing conditions [18,19]; therefore, the performance of printed biosensors depends not only on the intrinsic functionality of the chosen materials but also on how effectively they can be formulated and integrated within a given printing platform [20,21]. This review discusses recent advances in printed biosensors from the combined perspectives of printing technology and functional materials, with particular emphasis on the role of material design in enabling high-performance devices. First, the major printing techniques used in biosensor fabrication are summarized in regard to their material requirements and processing characteristics. The discussion then focuses on the key classes of nanomaterials employed in printed biosensors and how their structural and chemical properties contribute to their analytical performance. Finally, the current challenges and future directions are identified in relation to reliability, manufacturability, and practical translation.

## 2. Printing Technologies as Material Platforms for Printable Biosensor Fabrication

**Figure 1:**
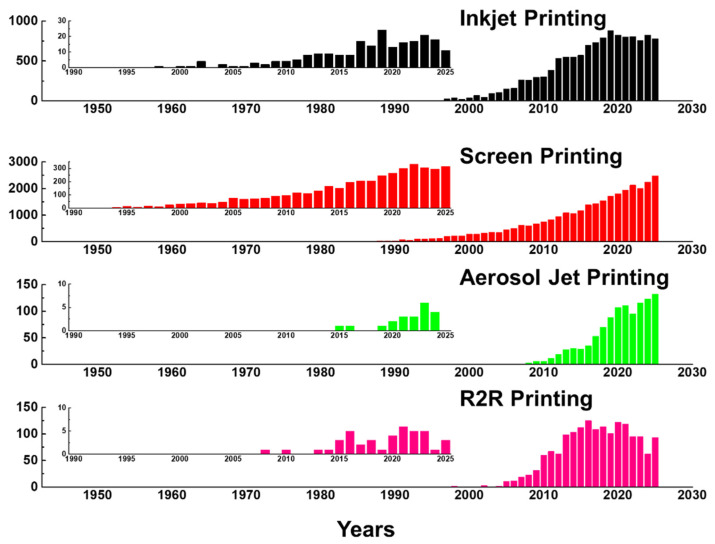
Annual publication trends for printed electronics (main graphs) and biosensor applications (insets) across the four major printing technologies: inkjet printing, screen printing, aerosol jet printing, and R2R printing. Data were obtained from Web of Science using the search terms “(printing technology name) AND (printed electronics OR biosensor)” for each respective category. Each panel is color-coded by printing technology for visual distinction.

**Figure 2:**
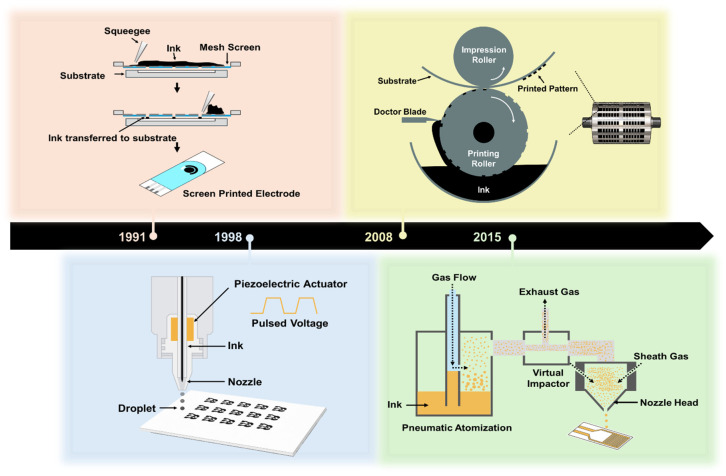
Schematic illustrations of the operating mechanisms of the four major printing technologies for biosensor fabrication. (**Top left**) Screen printing: a squeegee forces ink through a patterned mesh screen onto the substrate. (**Bottom left**) Inkjet printing: piezoelectric actuation ejects ink droplets through a nozzle in a drop-on-demand manner. (**Top right**) R2R gravure printing: ink is transferred from engraved cells on a printing roller to a moving substrate via an impression roller. (**Bottom right**) Aerosol jet printing: ink is atomized pneumatically and aerodynamically focused through a sheath gas flow for deposition. The timeline indicates the approximate periods when each printing technology was first applied to biosensor fabrication.

Printed biosensors are fabricated through the controlled deposition of functional inks onto substrates to form electrodes, sensing layers, and interconnects [22,23]. A wide range of printing technologies has been developed for biosensor fabrication, each exhibiting distinct characteristics in terms of resolution, printing speed, material compatibility, and scalability [24]. As shown in Figure 1, the number of publications related to inkjet printing, screen printing, aerosol jet printing, and R2R printing has increased substantially over the past few decades. This growth reflects several converging technological drivers: the expanding demand for rapid, low-cost, and portable point-of-care diagnostics [1,2,3]; the scalability and cost-effectiveness of printing-based fabrication relative to conventional photolithographic processes [4,5,6]; and the growing suitability of printed device formats for disposable and wearable health monitoring applications [7,8,9]. The insets, which display publication counts specifically for biosensor applications, reveal a similar upward trend that has accelerated from approximately 2015 onward, coinciding with the widespread adoption of flexible substrate technologies, the increased availability of functional nanomaterial inks, and heightened global demand for decentralized diagnostic solutions. Notably, screen printing shows the earliest and most sustained biosensor-specific growth, consistent with its established role in commercial glucose test strip manufacturing, whereas the more recent rise in inkjet and aerosol jet printing publications reflects growing interest in high-resolution and digital fabrication approaches for next-generation biosensor platforms. Figure 2 illustrates the operating mechanisms of these four printing technologies along with a timeline indicating their approximate adoption periods for biosensor applications. While these technologies are often classified based on their printing mechanisms or achievable feature sizes, their practical implementation in biosensor manufacturing is fundamentally governed by material-related constraints [25]. In particular, the rheological properties of nanomaterial inks, ink–substrate interactions, and the chemical and mechanical stabilities of functional materials under specific printing conditions play a decisive role in determining the printing fidelity, device performance, and manufacturing yield [26]. Consequently, the choice of printing technology cannot be decoupled from material selection and formulation, particularly for nanomaterial-based biosensors that integrate conductive, catalytic, and biological components within a single device architecture. This section reviews the major printing technologies employed for nanomaterial-based biosensor fabrication, including inkjet printing, screen printing, aerosol jet printing, and roll-to-roll (R2R) gravure printing, with emphasis on how each printing platform defines material requirements, processing windows, and design constraints for functional nanomaterials [27].

### 2.1. Inkjet Printing

#### 2.1.1. Operating Mechanism and Printing Performance

Inkjet printing is a non-contact, digital printing technique in which picoliter-scale droplets are selectively ejected from a nozzle and deposited onto a substrate in a drop-on-demand manner. Pattern formation is achieved without physical masks or contact with the substrate, allowing rapid design iterations and the efficient use of functional inks. Owing to these characteristics, inkjet printing has been widely adopted for prototyping and small- to medium-scale fabrication of printed electronics and biosensors.

Inkjet printing typically provides lateral feature resolutions in the range of 20–100 μm, primarily determined by nozzle diameter, droplet volume, and ink–substrate interactions. Under optimized conditions, silver conductive tracks with line widths as small as 40 μm have been reported [28,29]. Film thickness is governed by the ink concentration, drop spacing, and number of printing passes. Single-pass printing generally yields film thicknesses of approximately 100–500 nm, whereas multi-pass overprinting enables thickness accumulation to several micrometers. For example, Mypati et al. reported that approximately 22 printed layers, corresponding to a total thickness of ~28 μm, were required to approach the bulk resistivity of silver [30]. In terms of productivity, inkjet printing operates at moderate throughput, with typical printing speeds of 0.1–1 m/s, while maintaining material utilization efficiencies exceeding 90% [31]. These characteristics make inkjet printing well-suited for prototyping and low- to medium-volume production of biosensors. In industrial-scale manufacturing, throughput can be significantly enhanced through integration with R2R processing. Reported R2R inkjet systems have achieved web speeds of up to 10 m/min, while multi-nozzle industrial printheads combined with machine vision-based alignment systems enable registration accuracies better than 100 μm at printing speeds of 5 m/min, which is critical for multilayer biosensor architectures [32,33].

#### 2.1.2. Material Requirements and Selection for Inks and Substrates

Reliable inkjet printing requires precise control of the rheological properties of the ink to ensure stable droplet formation and accurate deposition [34]. Ink printability is commonly described by the dimensionless Ohnesorge number (Oh), which represents the ratio of the viscous forces to the inertial and surface tension forces. Stable inkjet printing is typically achieved within an Oh range of 0.1–1.0, corresponding to viscosities of 1–20 mPa·s and surface tensions of 28–35 mN/m for aqueous- and organic-solvent-based inks [22,35]. These rheological constraints directly govern the final electrochemical performance of the printed electrode: inks with viscosities below 1 mPa·s produce satellite droplets that result in non-uniform film deposition and increased electrode-to-electrode resistance variation, while inks exceeding 40 mPa·s cause nozzle clogging and irregular droplet spacing that reduce the electroactive surface area and elevate charge transfer resistance. Similarly, surface tension determines the contact angle on the substrate, which governs the final electrode geometry and edge definition; substrates with surface energy mismatched to the ink produce coffee-ring deposition patterns that concentrate conductive material at the electrode periphery, reducing the uniformity of the electron transfer pathway and introducing spatial variability in the amperometric response [26].

Material selection and formulation must also address printhead-related challenges. Satellite droplet formation can be reduced by incorporating polymers that enhance droplet cohesion during flight, whereas faceplate wetting caused by low-surface-tension inks can be mitigated by using appropriate surfactant formulations. In addition, the so-called “first drop problem,” which arises from solvent evaporation at the nozzle during idle periods, can be alleviated by the use of humectants or less volatile solvents [36]. For biosensor applications, ink formulations must additionally preserve biological activity and electrochemical functionality. Enzyme-containing inks require careful pH buffering, typically in the pH range of 6–8, and the inclusion of stabilizing agents such as trehalose, glycerol, or bovine serum albumin to maintain enzymatic activity during jetting and subsequent drying [37]. The high shear rates experienced during droplet ejection (10^4^–10^6^ s^−1^) can induce protein denaturation; therefore, surfactants are often incorporated at concentrations below the critical micelle concentration to reduce interfacial stress without compromising biomolecule integrity [38]. For conductive nanomaterial inks used in electrode fabrication, the particle size must be carefully controlled to prevent nozzle clogging, typically remaining below 1/50th of the nozzle diameter, while sufficient solid loading (10–40 wt.%) is maintained to achieve adequate post-sintering conductivity [38]. Carbon nanomaterial inks present additional challenges owing to their tendency to aggregate; effective dispersion strategies include covalent functionalization, surfactant stabilization, or polymer wrapping to produce stable colloidal suspensions that are compatible with inkjet deposition [34]. Post-printing thermal sintering is the most widely applied post-processing step for inkjet-printed conductive electrodes, directly governing device architecture and sensing performance. For silver nanoparticle inks on PET substrates, sintering at 120–150 °C for 30–60 min removes organic capping agents and promotes interparticle necking, reducing sheet resistance from >10^4^ Ω/sq to below 1 Ω/sq; however, temperatures exceeding 160 °C induce substrate warping and film cracking that compromise interlayer registration in multilayer biosensor architectures [28,30]. For biosensor applications where pre-immobilized biomolecules constrain the thermal budget, photonic sintering using intense pulsed light enables millisecond-scale processing at near-ambient substrate temperatures, achieving comparable conductivity gains while preserving biological activity—directly resolving the conflict between electrode performance and biorecognition layer stability that governs manufacturing reliability in inkjet-printed biosensors.

### 2.2. Screen Printing

#### 2.2.1. Operating Mechanism and Printing Performance

Screen printing is a well-established thick-film deposition technique that has been widely used for the fabrication of disposable electrochemical biosensors owing to its simplicity, low cost, and high reproducibility [39,40]. As a contact-based printing process, it enables sequential, layer-by-layer deposition of functional inks onto a broad range of substrates, resulting in mechanically robust electrode structures suitable for large-scale manufacturing. The long-standing commercial success of screen-printed electrodes (SPEs) in glucose biosensor strips demonstrates the industrial feasibility and technological maturity of this approach for point-of-care diagnostic applications [40].

Screen printing operates by forcing a viscous ink or paste through a patterned mesh screen onto a substrate using a squeegee [41]. The screen typically consists of a woven mesh, such as stainless steel or polyester, stretched over a rigid frame and combined with a photolithographically defined stencil that blocks ink transfer in non-printing regions [42]. During printing, the squeegee traverses the screen surface under controlled pressure, bringing the mesh into contact with the substrate and driving the ink through the open mesh areas. After the squeegee passes, the screen separates from the substrate (snap-off), leaving behind the printed pattern. Screen printing is commonly implemented in flatbed and rotary configurations. Flatbed screen printing employs a stationary screen with a moving squeegee, providing high patterning accuracy suitable for laboratory-scale and low-volume production. In contrast, rotary screen printing utilizes a cylindrical screen that rotates continuously over a moving substrate, enabling high-speed R2R processing with web speeds typically ranging from 5 to 20 m/min for industrial-scale manufacturing [43,44].

In terms of printing performance, screen printing typically achieves lateral feature resolutions of 50–200 μm, governed primarily by the mesh count, wire diameter, and paste rheology [45]. Higher mesh counts (300–500 threads per inch) combined with finer wire diameters (15–25 μm) enable line widths approaching 30–50 μm, while advanced stainless-steel screens have demonstrated electrode features as narrow as 40 μm for interdigitated biosensor architectures [46,47]. A notable advantage of screen printing is its thick-film capability, with single-pass printing yielding film thicknesses in the range of 5–100 μm. This is particularly advantageous for electrochemical biosensors where electrode conductivity and the electrochemically active surface area directly influence sensing performance [1,48]. Moreover, screen printing offers excellent scalability from laboratory prototyping to industrial mass production [49]. Integration with rotary screen printing and R2R processing has enabled the commercial manufacture of billions of glucose test strips annually, underscoring the industrial robustness and scalability of this printing technology [50,51,52].

#### 2.2.2. Material Requirements and Selection for Inks and Substrates

Screen-printing inks, commonly referred to as pastes, require substantially higher viscosities than inkjet inks to prevent spontaneous ink flow through the mesh under gravity. Typical paste viscosities range from 1 to 100 Pa·s, and pastes must exhibit strong shear-thinning (pseudoplastic) behavior to ensure successful printing [53]. Under the high-shear conditions imposed by the squeegee, the paste must flow readily through the mesh openings, followed by rapid viscosity recovery after deposition to prevent pattern spreading and maintain edge definition. This thixotropic behavior is critical for achieving high-resolution printed features [54].

Screen printing pastes generally comprise three primary components: functional particles (e.g., carbon, silver, gold, platinum, and graphite), polymeric binders (e.g., ethyl cellulose, polyvinyl butyral, and acrylic resins), and solvents (e.g., terpineol, texanol, and glycol ethers) [55,56]. Paste rheology is commonly described using the Herschel–Bulkley model, with optimized yield stress values typically in the range of 5–50 Pa depending on the mesh parameters and squeegee conditions [57]. The quantitative relationship between paste rheology and electrochemical performance operates through two primary mechanisms: first, yield stress governs the degree of post-deposition spreading, which determines the final electrode thickness and uniformity—pastes with insufficient yield stress spread beyond the intended pattern boundaries, reducing electrode definition and increasing inter-electrode cross-talk in multiplexed biosensor arrays; second, the shear-thinning index of the paste controls ink transfer efficiency through the mesh, with sub-optimal flow behavior leading to incomplete mesh emptying, pin-hole defects in the deposited film, and consequent increases in the apparent charge-transfer resistance measured by electrochemical impedance spectroscopy [53,57]. For electrochemical biosensor applications, material selection must balance printability with electrochemical performance. Carbon-based pastes dominate SPE fabrication owing to their low cost, wide electrochemical-potential window, and chemical inertness; however, the choice of carbon allotrope strongly affects sensor behavior. Graphite-based pastes provide good electrical conductivity but a relatively low surface area, whereas carbon black (CB) offers a higher surface area at the expense of an increased paste viscosity. The incorporation of carbon nanomaterials, such as carbon nanotubes or graphene, can significantly enhance the electron transfer kinetics and effective surface area; however, careful dispersion strategies are required to prevent aggregation, which compromises both printability and electrochemical reproducibility [58].

Binder selection plays a critical role in determining the rheological properties and electrochemical performance of biosensors. Excessive binder content improves print fidelity but introduces insulating barriers that hinder electron transfer, whereas insufficient binder results in poor adhesion and mechanical instability of the printed electrodes [59]. For enzyme-modified SPEs, additional challenges arise from the sensitivity of biomolecules to organic solvents commonly used in screen printing pastes. Direct incorporation of enzymes into carbon pastes can lead to the loss of biological activity, motivating alternative strategies such as post-printing enzyme immobilization or the use of water-based binder systems compatible with biomolecules [60]. For mediator-based first-generation biosensors, careful optimization of the mediator loading and spatial distribution within the paste is required to ensure efficient electron shuttling while minimizing mediator leaching during sensor operation [61].

Screen printing is compatible with a broad range of rigid and flexible substrates owing to its contact-based deposition mechanism and tolerance to high-viscosity pastes. Polymeric substrates such as polyethylene terephthalate (PET) [62] and polyimide (PI) [63] are widely used as disposable biosensors because of their low cost and mechanical flexibility. Ceramic substrates such as alumina offer superior thermal stability and chemical resistance in high-temperature curing processes [64]. Paper substrates have emerged as attractive platforms for ultra-low-cost and environmentally friendly biosensors [65], whereas textile substrates enable the fabrication of wearable biosensors for continuous health monitoring applications [66]. Post-printing thermal curing in screen printing typically involves treatment at 60–120 °C to evaporate residual solvents, crosslink polymeric binders, and consolidate the printed film, with direct consequences for device architecture and electrochemical performance. Undercuring results in mechanically fragile films with residual solvent that increases contact resistance and impairs long-term sensing stability, whereas overcuring densifies the binder matrix, reduces accessible electrode porosity, and lowers the electroactive surface area available for analyte interaction—thereby degrading sensitivity [59]. For enzyme-containing layers deposited after electrode fabrication, post-printing curing must be limited to mild conditions (≤60 °C) to preserve biological activity, necessitating separate curing protocols for electrode and biorecognition layers and imposing a fundamental constraint on manufacturing throughput that represents one of the principal challenges for manufacturing reliability in screen-printed biosensor production.

### 2.3. Aerosol Jet Printing

#### 2.3.1. Operating Mechanism and Printing Performance

Aerosol jet printing (AJP) is a non-contact, direct-write additive manufacturing technique that has garnered massive attention for the fabrication of high-resolution printed electronics and biosensors. AJP uniquely combines microscale resolution, broad ink compatibility, and an exceptional conformal printing capability on complex three-dimensional surfaces, positioning it as a promising platform for biosensor applications requiring precise patterning of functional materials on non-planar substrates.

The AJP process comprises four main steps: atomization of the ink, densification of the aerosol stream, aerodynamic focusing, and deposition onto the substrate [24]. Ink atomization can be achieved using either ultrasonic or pneumatic mechanisms. Ultrasonic atomization employs piezoelectric transducers operating at frequencies of 1.5–2.5 MHz to generate aerosol droplets with diameters of 1–5 μm and is suitable for low- to moderate-viscosity inks (1–10 cP) with minimal material wastage [67]. In contrast, pneumatic atomization uses high-velocity nitrogen gas to shear ink into droplets, accommodating significantly higher viscosities (up to 1000 cP); however, it requires virtual impactor filtration to remove oversized droplets [68,69,70]. Following atomization, the aerosol stream is aerodynamically focused by a coaxial sheath gas flow, which compresses the aerosol plume to approximately one-tenth of the nozzle diameter. This aerodynamic focusing enables the deposition of fine features as small as 10 μm using nozzles with diameters of 100–300 μm [71,72,73]. A distinctive feature of the AJP is its large standoff distance, typically ranging from 1 to 5 mm and extendable beyond 10 mm, which allows conformal printing on curved, textured, and stepped surfaces without nozzle adjustments [74,75]. As a result, AJP enables reliable deposition on three-dimensional substrates with surface inclinations of up to 70°, significantly expanding the accessible design space compared with conventional inkjet printing [75,76].

In terms of printing performance, AJP achieves lateral feature resolutions of 10–100 μm under standard conditions and as fine as 5–6 μm with optimized process parameters, corresponding to approximately 2–4 times higher resolution than inkjet printing [77,78]. The resolution is governed by the atomizer type, sheath-to-carrier gas-flow ratio (typically optimized between 1.5 and 3.0), nozzle geometry, standoff distance, and ink properties [79,80]. Single-pass deposition generally yields film thicknesses of 100–500 nm, whereas multi-pass deposition enables thickness accumulation up to several micrometers. This layer-by-layer capability allows the fabrication of complex microstructures, such as pillar arrays, microfluidic channels, and sample confinement features integrated with sensor electrodes [81,82]. From a manufacturing perspective, the AJP occupies a niche between laboratory-scale prototyping and low-volume production. Typical printing speeds range from 1 to 10 mm/s for high-resolution patterning and can be increased to approximately 50 mm/s when the resolution requirements are relaxed [83,84]. Although serial, vector-based deposition inherently limits throughput relative to area-parallel printing techniques, recent developments, such as wide-flow AJP using rectangular nozzles, generate planar aerosol jets with millimeter-scale widths, substantially improving throughput while maintaining a submicron vertical resolution [85].

#### 2.3.2. Material Requirements and Selection for Inks and Substrates

A key advantage of AJP is its exceptionally broad ink compatibility, accommodating viscosities spanning approximately 1–1000 cP, which is nearly 50 times wider than that of inkjet printing [24]. This wide processing window enables the deposition of diverse functional materials, including metallic nanoparticles, carbon nanomaterials, polymers, ceramics, and biological materials, within a single printing platform. For ultrasonic atomization, optimal ink viscosities typically range from 1 to 10 cP, with maximum particle sizes below 50 nm, whereas pneumatic atomization relaxes these constraints to viscosities up to 1000 cP and particle sizes up to approximately 500 nm [86,87]. Surface tensions in the range of 30–50 mN/m are generally targeted to achieve stable aerosol formation and controlled deposition [88]. The sheath-to-carrier gas flow ratio—typically optimized between 1.5 and 3.0—functions as the primary rheological control parameter in AJP, governing aerosol plume width and deposition linewidth independently of ink viscosity [79,80]. At sub-optimal flow ratios, overspray deposits conductive material outside the intended electrode boundaries, effectively increasing the electrode geometric area without a proportional increase in the electroactive surface area, thereby reducing the normalized sensitivity of the biosensor. Conversely, excessive sheath gas flow produces narrow, dense lines with elevated film stress that can crack during post-processing or mechanical deformation, increasing contact resistance and degrading long-term sensing stability.

Solvent selection plays a critical role in determining the print quality and pattern fidelity. High-volatility solvents can lead to premature evaporation, aerosol dispersion, and overspray, whereas the addition of 5–10 vol.% low-volatility co-solvents such as terpineol or ethylene glycol has been shown to improve line continuity and morphology [89,90,91]. For biosensor fabrication, the broad material compatibility of AJP provides distinct advantages for the integration of heterogeneous functional layers. In particular, ultrasonic atomization subjects inks to lower shear stresses than inkjet nozzle ejection, which can be beneficial for preserving the activity of sensitive biomolecules [68]. However, the aerosolization process exposes inks to large gas–liquid interfacial areas, which may induce protein denaturation. Consequently, protective additives such as polyethylene glycol, Pluronic surfactants, or protein stabilizers are often required for enzyme-containing inks to maintain biological functionality [92].

Carbon nanomaterial inks benefit significantly from the tolerance of AJP to higher viscosities, allowing increased solid loading and improved electrical conductivity without the nozzle-clogging issues commonly encountered in inkjet systems [93]. For multilayer biosensor fabrication, AJP enables the sequential deposition of electrode materials, dielectric layers, and biorecognition elements with high spatial precision. However, ink–ink compatibility must be carefully considered to prevent the dissolution, intermixing, or delamination of previously deposited layers during subsequent printing steps [94]. This extended standoff distance further allows deposition onto pre-assembled sensor architectures, facilitating the integration of AJP-deposited biorecognition layers with conventionally manufactured electrode platforms [81]. AJP is compatible with a wide range of rigid and flexible substrates, including glass, silicon, ceramics, FR-4 printed circuit boards, PI films for high-temperature processing, PET for low-cost applications, paper, textiles, and elastomeric substrates such as polydimethylsiloxane for flexible and stretchable biosensors [71,95,96,97,98,99]. Unlike screen and inkjet printing, AJP deposits thin, low-mass films that frequently require post-processing to achieve target electrical and mechanical properties, with each step inducing distinct changes to device architecture and sensing performance. Photonic sintering and low-temperature annealing (80–150 °C) are commonly applied to metallic nanoparticle AJP films to remove residual solvent and surfactant residues, reducing sheet resistance and improving film adhesion to the substrate without damaging flexible or elastomeric substrates [77,88]. For carbon nanomaterial-based AJP films, thermal annealing in inert atmospheres restores electrical conductivity disrupted during aerosolization, while simultaneously tuning surface roughness and porosity—parameters that directly govern subsequent biomolecule immobilization density and electron transfer kinetics at the sensing interface. In multilayer AJP biosensor architectures, the sequence and conditions of post-processing steps must be carefully controlled to prevent solvent-induced swelling or delamination of previously deposited layers, making post-processing a critical determinant of interlayer registration accuracy and manufacturing reliability.

### 2.4. R2R Printing

#### 2.4.1. Operating Mechanism and Printing Performance

R2R printing technologies represent continuous manufacturing platforms designed for high-throughput, large-area fabrication on flexible substrates. Unlike sheet-fed or serial deposition techniques, R2R processes transport flexible webs from supply rolls through sequential processing stations, including printing, drying or curing, and surface treatment, and collect the processed material on the output rolls [100]. Originally developed for the graphic arts and packaging industries, R2R printing has been increasingly adopted for printed electronics and biosensor manufacturing owing to its exceptional scalability and cost-effectiveness at the production scale. Among the R2R techniques, gravure printing is the most widely employed method for biosensor fabrication and is the focus of this section.

A typical R2R gravure printing system consists of an unwinder, printing and processing units, drying or curing modules, and a rewinder, with precise control of web tension throughout the process [101,102]. Gravure printing involves four sequential stages: ink cell filling, doctoring (wiping), ink transfer, and spreading [102]. In this process, an engraved printing cylinder rotates while partially immersed in an ink fountain, allowing the recessed cells to be filled with ink. A doctor blade then removes excess ink from the non-imaged areas, leaving the ink confined within the engraved cells [5]. During printing, the substrate is pressed against the ink-filled gravure cylinder using an impression roller, enabling ink transfer from the cells to the substrate through a combination of capillary forces, applied pressure, and surface energy matching. Multi-station gravure configurations allow the sequential deposition of multiple functional layers within a single R2R pass, facilitating multilayer device fabrication [103,104].

In terms of printing performance, gravure printing typically achieves lateral feature resolutions of 50–200 μm under standard industrial conditions using engraving densities of 100–200 lines per inch [104,105]. With optimized engraving and process control, line widths of approximately 20 μm have been demonstrated, while ultra-precision diamond micro-engraving has enabled cell widths as small as 7 μm, allowing sub-10-μm printed features [106]. Film thickness is primarily governed by cell depth and transfer efficiency and generally ranges from 0.2 to 5 μm per pass [106]. A notable advantage of gravure-based R2R printing is its exceptionally high throughput. While publication printing systems operate at speeds of 100–1000 m/min, printed electronics and biosensor manufacturing typically employ web speeds of 5–20 m/min to ensure adequate ink transfer, drying, and registration accuracy [107,108]. Demonstrated R2R gravure processes include continuous printing over 150 m PET webs with uniform electrochemical performance and fully gravure-printed thin-film transistor arrays fabricated at 8 m/min. Device yields exceed 98%, highlighting the industrial maturity and scalability of this technology [109].

#### 2.4.2. Material Requirements and Selection for Inks and Substrates

Gravure printing accommodates a moderate viscosity window compared with other printing techniques, typically ranging from 10 to 500 cP [109,110]. Lower viscosities (10–100 cP) are preferred for high-resolution patterning to ensure efficient cell filling and clean doctoring, whereas higher viscosities (100–500 cP) are preferred for thick-film deposition. For biosensor electrode fabrication, conductive inks commonly employ viscosities in the range of 50–200 cP to balance cell filling, ink-transfer efficiency, and post-transfer spreading. The surface tension must be carefully optimized to ensure stable printing. Excessively low surface tension leads to uncontrolled spreading and poor edge definition, while a high surface tension impedes complete cell filling and ink release from the gravure cylinder [111]. Unlike inkjet printing or AJP, gravure printing subjects inks to high shear rates during doctoring, up to approximately 10^5^ s^−1^, as well as compressive forces within the printing nip, thereby imposing stringent material robustness requirements [112]. The viscosity of the gravure ink at this high shear rate is the primary determinant of ink transfer efficiency from the engraved cell to the substrate: inks with insufficiently low dynamic viscosity under high shear fail to release cleanly from the cell, resulting in incomplete ink transfer, reduced film thickness, and lower electrode conductivity than predicted from the cell volume. Conversely, excessively low low-shear viscosity causes uncontrolled spreading after cell release, widening the printed line beyond the cell geometry and reducing electrode definition. In biosensor electrode fabrication, this viscosity window directly controls the final film thickness (0.5–12 µm per pass), which governs both the sheet resistance of the conductive layer and the diffusion pathway length for analytes accessing the electroactive surface—parameters that quantitatively determine the amperometric sensitivity and response time of the finished sensor [109,112].

For conductive electrode inks, these mechanical stresses typically pose minimal concern. However, direct gravure printing of enzyme-containing inks results in a substantial loss of biological activity, often exceeding 50%, owing to mechanical and interfacial stresses [38,113]. Consequently, gravure printing is rarely used for direct deposition of biorecognition elements. Instead, hybrid manufacturing approaches are commonly employed, in which gravure printing is used to fabricate electrode structures and underlayers, followed by inkjet printing or drop-casting of biomolecules [114].

Carbon nanomaterial inks present additional challenges for gravure printing. The doctoring process can induce aggregation or morphological damage to nanomaterials, potentially degrading their electrical performance and print uniformity. Therefore, optimized binder systems, a controlled doctor blade pressure, and tailored solvent compositions are required to maintain dispersion stability while preserving print quality [115]. Maintaining ink stability during extended R2R production runs is a critical requirement. Solvent evaporation from the ink fountain can induce viscosity drift during long printing sessions, necessitating active ink replenishment systems or the use of low-volatility solvent formulations to ensure consistent printability over hundreds of meters of substrate [115].

Substrate selection is equally critical in R2R biosensor manufacturing. Gravure printing exhibits broad compatibility with flexible substrates, among which PET is the most widely used because of its favorable mechanical properties, optical clarity, chemical resistance, and cost-effectiveness [116]. For processes requiring elevated curing or sintering temperatures above 150 °C, PI [117] and polyethylene naphthalate [118] substrates offer superior thermal stability. Paper substrates have also emerged as attractive platforms for ultra-low-cost, disposable biosensors, although their dimensional stability and moisture sensitivity must be carefully managed. Finally, the drying and curing conditions strongly influence both material performance and dimensional stability in the R2R processes. Non-uniform thermal gradients can induce web-tension fluctuations, leading to registration errors that are particularly detrimental in multilayer biosensor architectures [119]. Furthermore, post-printing hydrothermal treatment—employed to grow functional nanostructures such as ZnO nanorods on R2R-printed seed layers—introduces additional dimensional changes and surface chemistry modifications that must be decoupled from the electrode fabrication step to prevent substrate deformation and maintain registration accuracy across subsequent printing passes. Therefore, integrated process control of ink formulation, drying kinetics, post-treatment conditions, and web handling is essential to fully exploit the advantages of R2R gravure printing for biosensor manufacturing, and represents one of the most practically important determinants of manufacturing reliability at production scale.

The printing technologies reviewed in this section each offer distinct combinations of resolution, throughput, material compatibility, and manufacturing scalability, and no single technique universally satisfies all requirements for biosensor fabrication. Table 1 and Table 2 summarize the key technical parameters, advantages, and limitations of inkjet printing, screen printing, AJP, and R2R gravure printing. Collectively, these comparisons highlight that the selection of an appropriate printing technology must be guided by the specific requirements of the target biosensor application, including the desired feature resolution, production volume, functional material characteristics, and cost constraints. Furthermore, hybrid manufacturing strategies that combine multiple printing techniques within a single fabrication workflow are increasingly employed to leverage the complementary strengths of each method while mitigating their individual limitations. Beyond these technical parameters, the four platforms differ substantially in their commercial and technological maturity. Screen printing has achieved the highest readiness for biosensor applications, as demonstrated by its deployment in the mass production of glucose test strips at billions of units annually and in lateral flow immunoassay components [50,51,52]; its commercial success is enabled by a well-established quality control infrastructure and the compatibility of its thick-film capability with simple electrode configurations suited to disposable diagnostics. R2R gravure printing has demonstrated pilot-scale electrode production with device yields exceeding 98% [109], but integration of biorecognition layers within continuous high-shear R2R workflows remains unresolved at production scale. Inkjet and aerosol jet printing are primarily at the prototyping and near-clinical validation stage, with batch-to-batch reproducibility and equipment cost representing the principal barriers to commercial deployment. Shared barriers across all platforms include the absence of standardized ink characterization protocols, insufficient long-term stability data for biorecognition layers under real-world conditions, and regulatory pathway complexity under frameworks.

## 3. Functional Nanomaterials for Printed Biosensor Fabrication

**Figure 3:**
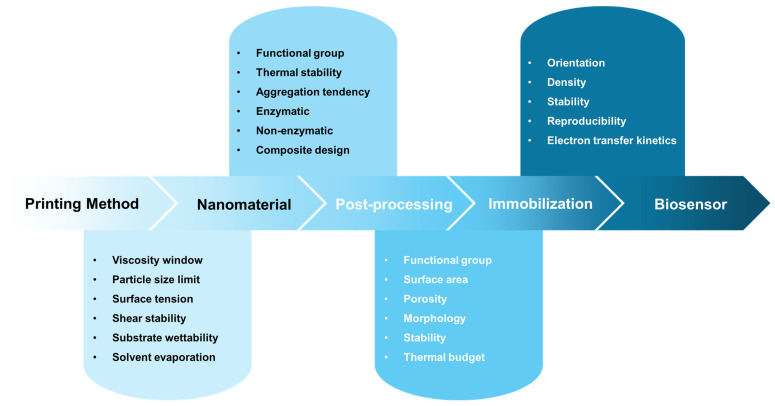
Integrated co-design framework for printed biosensor fabrication. The diagram illustrates the sequential and interdependent relationships among five key stages: printing method selection, nanomaterial selection, post-processing, immobilization strategy, and biosensor performance. The lower panels identify the critical ink-related parameters (viscosity window, particle size limit, surface tension, shear stability, substrate wettability, and solvent evaporation rate) that printing method selection imposes on ink formulation, and the material surface parameters (functional group type, electroactive surface area, film porosity and morphology, operational stability, and thermal budget) that post-processing conditions make available or restrict for subsequent immobilization. The upper panels summarize the principal nanomaterial design considerations that determine compatible post-processing routes (functional group availability, thermal stability, aggregation tendency, enzymatic versus non-enzymatic sensing configuration, and composite design requirements), and the immobilization outcomes that collectively govern analytical performance (biomolecule orientation and surface density, long-term binding stability, electron transfer kinetics, and batch reproducibility).

The selection of a functional nanomaterial for a printed biosensor cannot be made independently of the printing platform, post-processing conditions, or immobilization strategy. Rather, each upstream design decision constrains the available options at subsequent stages, making printed biosensor development an inherently co-design challenge. Figure 3 summarizes this interdependence as a sequential framework in which printing method selection imposes rheological and particle-size constraints on ink formulation; ink formulation determines the compatible nanomaterial class and its surface chemistry; the nanomaterial’s thermal stability and surface functional group profile define the permissible post-processing routes; and post-processing conditions in turn enable or restrict the immobilization strategies available for biorecognition element attachment. The cumulative outcome of these interdependent decisions determines the analytical performance of the biosensor, including sensitivity, operational stability, and batch reproducibility. The following sections discuss each nanomaterial class—carbon-based nanomaterials (Section 3.1), metal-oxide nanomaterials (Section 3.2), metal nanoparticles (Section 3.3), conductive polymers (Section 3.4), and dielectric materials (Section 3.5)—within this co-design framework.

### 3.1. Carbon-Based Nanomaterials

Carbon-based nanomaterials are among the most widely used material platforms for printed electrochemical biosensors because of their high electrical conductivity, tunable surface chemistry, and compatibility with solution-based processing. Materials such as CB, carbon nanotubes (CNTs), graphene-based materials (including graphene oxide and reduced graphene oxide), and carbon quantum dots (CQDs) have been explored to enhance electron transfer, increase the electroactive surface area, and enable the stable immobilization of biorecognition elements. These complementary properties have significantly improved the sensitivity, selectivity, and practical applicability of printed biosensors. This section summarizes the major classes of carbon nanomaterials and their roles in enhancing biosensor performance for point-of-care diagnostics.

CB is one of the most widely used and cost-effective carbon nanomaterials for printed electrodes, serving as the primary conductive filler in commercial screen-printed electrode systems owing to its low cost, facile synthesis, and excellent compatibility with polymeric binders. However, the relatively low surface area and limited electrocatalytic activity of pristine CB have motivated the development of hybrid architectures. The incorporation of catalytic mediators such as Prussian blue nanoparticles enhances hydrogen peroxide detection by providing additional redox-active sites while preserving the electrochemical characteristics of the electrode [120], and porous materials such as copper-based metal–organic frameworks introduce synergistic electron transfer pathways that extend CB-based sensing to targets such as nitrite with nanomolar sensitivity [121]. Beyond environmental monitoring, CB-based inks have demonstrated versatility across biosensing applications ranging from wearable enzymatic glucose detection in sweat [122] to immunosensing of SARS-CoV-2 on flexible substrates [123] and electrochemical detection of neurodegenerative disease biomarkers [124,125]. These examples collectively illustrate that the primary value of CB in printed biosensors lies not in its intrinsic electrochemical performance, but in its role as a printable, processable scaffold that enables the integration of more functionally specific components—a design principle that distinguishes it from the higher-performance but more challenging-to-process carbon nanomaterials discussed in the following sections.

CNTs provide significantly improved electrical conductivity, high aspect ratios, and large accessible surface areas compared with CB, making them attractive materials for printed biosensors. However, their strong van der Waals interactions and hydrophobic surfaces present challenges for their dispersion and biomolecule immobilization. Therefore, various functionalization and dispersion strategies have been developed to improve their processability in printable inks. Covalent functionalization via oxidative acid treatment (e.g., HNO_3_/H_2_SO_4_) introduces carboxyl (–COOH) and hydroxyl (–OH) groups on the CNT sidewalls, enabling EDC/NHS-mediated coupling of enzymes and antibodies with high stability; however, this process partially disrupts the sp^2^ carbon lattice, reducing electrical conductivity and electron transfer kinetics. Non-covalent strategies—including π–π stacking of pyrene-based linkers and polymer wrapping with Nafion or chitosan—preserve the intrinsic conductivity of CNTs while providing anchoring sites for biomolecules, and are therefore preferred for electrochemical biosensor applications where electron transfer kinetics are critical. For example, Nafion has been widely employed as both a dispersant and selective membrane to stabilize CNT suspensions while suppressing interference from negatively charged species such as ascorbic acid, thereby enabling the sensitive electrochemical detection of dopamine in complex biological matrices [126]. In addition to laboratory-scale demonstrations, scalable fabrication strategies have been explored for CNT-based devices. R2R gravure printing of single-walled CNT active layers on flexible PET substrates has demonstrated high-throughput production with device yields exceeding 98%, highlighting the potential for the industrial-scale manufacturing of CNT-based sensing platforms [109]. CNT nanocomposites incorporating catalytic nanoparticles or conductive polymers have further enhanced the biosensor performance. For example, Multi-walled carbon nanotube (MWCNT)-based nanocomposites combined with platinum nanoparticles and PEDOT/Prussian blue-modified electrodes have enabled sensitive glucose detection over clinically relevant concentration ranges with excellent operational stability [127]. In these ternary architectures, each component fulfills a distinct functional role that is unachievable by any single material alone: MWCNTs serve as a high-aspect-ratio conductive scaffold that reduces interfacial charge-transfer resistance and increases the electroactive surface area; Pt nanoparticles anchored on the MWCNT surface provide electrocatalytic activity toward H_2_O_2_ oxidation at low overpotential; and the PEDOT/Prussian blue layer acts as an electron mediator that lowers the operating potential and suppresses electrochemical interference from co-existing species such as ascorbic acid and uric acid. The high surface area of the CNT scaffold also prevents Pt nanoparticle agglomeration during repeated electrochemical cycling, maintaining catalytic activity over extended operation—a stability benefit that neither Pt nanoparticles nor PEDOT films alone can provide. Functionalized MWCNT/Ag nanoparticle composites have also been reported for non-enzymatic dopamine detection, where surface functional groups improve aqueous dispersibility and provide anchoring sites for metal nanoparticles [128]. In addition, CNT-modified printed electrodes have been applied to non-invasive biomarker monitoring and cell-based sensing platforms. MWCNT electrodes with immobilized uricase have enabled the selective detection of uric acid in saliva for non-invasive diagnostics [129], whereas Au nanoparticle (NP)/MWCNT/Nafion-modified electrodes have been used to monitor dopamine release from living cells, demonstrating the potential of CNT-based biosensors for drug screening and cellular analysis [130].

Graphene and its derivatives, including graphene oxide and reduced graphene oxide (rGO), have been widely explored for printed biosensors owing to their high theoretical surface area (~2630 m^2^·g^−1^), excellent electrical conductivity, and mechanical flexibility. In practice, oxidized derivatives are frequently employed because their oxygen-containing functional groups (epoxide, hydroxyl, carboxyl, and carbonyl groups) improve dispersibility and facilitate biomolecule immobilization while maintaining sufficient electrical conductivity for electrochemical sensing. Specifically, carboxyl groups on graphene oxide surfaces serve as EDC/NHS coupling sites for amine-containing biomolecules such as antibodies and enzymes, whereas epoxide groups can react directly with amine residues under mild alkaline conditions. Electrochemical reduction of graphene oxide after immobilization partially restores conductivity while retaining surface functional groups, enabling a favorable balance between electron transfer efficiency and immobilization capacity that is difficult to achieve with pristine graphene. Graphene-based inks have been integrated into various printed biosensor architectures. Inkjet-printed graphene electrodes combined with conductive polymers, such as PEDOT:PSS, have enabled low-cost disposable glucose sensors on paper substrates suitable for resource-limited settings [131]. In transistor-based platforms, graphene-gated organic electrochemical transistors have demonstrated enhanced signal amplification and improved detection sensitivity compared with conventional amperometric sensors [132]. Graphene oxide and its derivatives are frequently employed as platforms for immobilizing bio-recognition elements, including antibodies, enzymes, and aptamers, thereby providing target selectivity in biosensor systems. For example, electrochemically reduced graphene oxide has served as an effective platform for antibody immobilization and signal amplification, enabling the simultaneous detection of cortisol and lactate with detection limits of 0.1 ng·mL^−1^ and 0.1 mM, respectively [133]. Advances in printing techniques have expanded the application of graphene-based biosensors. Aerosol jet printing enables high-resolution electrode fabrication, allowing graphene-based immunosensors for cytokine detection with limits of detection as low as 25–46 pg·mL^−1^ while maintaining mechanical stability under repeated bending [81]. Flexible graphene inks prepared for stencil printing have also supported enzymatic glucose sensors with sensitivities of approximately 22.01 μA·mM^−1^·cm^−2^ [14]. Laser-induced graphene (LIG) has recently emerged as an alternative fabrication strategy that directly converts polymer substrates such as PI into porous graphene structures through laser irradiation, eliminating the need for ink formulation. LIG electrodes decorated with conductive polymers and metal nanoparticles have demonstrated high-performance glucose sensing with sensitivities exceeding 300 μA·mM^−1^·cm^−2^ and submicromolar detection limits [134]. LIG-based platforms have also enabled the development of multianalyte sensing systems. For example, LIG biosensors for simultaneous glucose and lactate detection have demonstrated sensitivities of 41.1 and 12.4 μA·mM^−1^·cm^−2^ with detection limits of 14.9 μM and 2.4 mM, respectively, while maintaining individual sensor performance after integration [135]. Hybrid LIG systems incorporating conductive polymer inks, such as PEDOT:PSS or polyaniline (PANI), have further improved sensing performance; graphene–PEDOT:PSS- and graphene–PANI-modified LIG electrodes have been used for dopamine and interleukin-6 detection, showing high reproducibility (Relative Standard Deviation = 0.76%), mechanical stability under repeated bending (RSD = 4.39% after 15 bends), and selectivity approximately 41-fold higher than conventional screen-printed electrodes [136]. The performance advantage of these hybrid systems arises from a synergistic division of roles: the porous LIG network provides a three-dimensional conductive scaffold with high surface area and mechanical resilience under bending, while the overcoated conductive polymer layer introduces electrochemical selectivity through its redox-active amine or sulfonate functional groups that interact preferentially with target analytes. This combination overcomes the selectivity limitation of bare LIG electrodes without sacrificing the flexibility and substrate-integrated fabrication that distinguish LIG from ink-based printing approaches. In addition, paper-derived LIG electrodes have been explored as environmentally sustainable platforms, enabling mediated enzymatic glucose sensing with detection limits of ~50–75 μM using ferrocene-carboxylic acid as an electron mediator [137].

CQDs and graphene quantum dots (GQDs), typically smaller than 10 nm, represent nanoscale carbon materials with high surface-to-volume ratios, abundant edge sites, and quantum-confined electronic properties. These structural features enable efficient enzyme immobilization and enhanced electrochemical activity on printed electrode platforms. The abundant edge-site functional groups on GQDs—particularly carboxyl and amine groups introduced via surface doping or chemical treatment—support both covalent coupling (EDC/NHS, glutaraldehyde crosslinking) and affinity-based immobilization (streptavidin–biotin, protein A/G), providing a versatile immobilization scaffold in a nanoscale footprint that is well-suited to the high-density electrode architectures of printed biosensors. For example, GQD-based nanocomposites have demonstrated strong signal amplification in nucleic acid sensing. GQD–gold nanoparticle-modified screen-printed electrodes have enabled femtogram-level detection of HIV RNA through probe DNA immobilization on L-cysteine-functionalized GQD surfaces [138]. Recent advances in green synthesis strategies have further improved the properties of GQDs. Nitrogen-doped GQDs synthesized through pyrolysis-free routes have achieved high doping levels (~14%) and enhanced electrochemical performance, enabling the ultrasensitive detection of small biomolecules such as histidine at concentrations as low as 0.01 nM on screen-printed electrodes [139].

The representative performance metrics of carbon-nanomaterial-based printed biosensors are summarized in Table 3, highlighting how material selection, processing strategies, and device architectures collectively influence analytical performance. The Printing Method column in Table 3 reveals a clear pattern in material–process compatibility: CB-based composites are exclusively fabricated by screen printing, consistent with their high-viscosity paste formulations and thick-film deposition requirements; CNT-based systems employ screen printing for high-solid-loading paste electrodes but shift to inkjet printing and R2R gravure for thin-film and scalable applications; graphene-based systems span the broadest range of printing techniques—inkjet, screen, stencil, and laser-induced fabrication—reflecting the versatility of graphene ink formulations across viscosity ranges; and GQD-based sensors rely predominantly on screen printing for their application on standard carbon SPE platforms. Early printed electrodes primarily relied on CB as a conductive filler, whereas more advanced nanostructured materials, such as CNTs, graphene derivatives, and quantum dots, provide enhanced electron transport, increased electroactive surface area, and improved interfaces for biomolecule immobilization. These materials offer complementary functionalities, including efficient charge transfer through one-dimensional nanostructures, high surface area and signal amplification in graphene-based systems, and nanoscale scaffolds for enzyme or nucleic acid recognition in quantum dot architectures. Importantly, the surface chemistry of each carbon nanomaterial dictates the applicable immobilization strategy: CNTs favor non-covalent π–π stacking or polymer-wrapping approaches to preserve conductivity; graphene oxide and rGO exploit oxygen-containing functional groups for EDC/NHS covalent coupling of antibodies and enzymes; and GQDs enable surface functionalization via thiol or amine chemistry on heteroatom-doped edge sites. The choice of immobilization strategy thus represents an additional design variable that must be co-optimized alongside material selection and printing process to achieve the target biosensor performance. Overall, carbon-based nanomaterials offer the broadest design space of any material class reviewed here, spanning from the industrial-scale processability of CB to the ultrasensitive nucleic acid detection enabled by GQDs. Their principal limitations are aggregation during ink formulation, sensitivity of electrochemical performance to oxidation state and defect density, and the need for functionalization strategies that balance immobilization stability against conductivity preservation. From a practical standpoint, screen-printed CB-based electrodes remain the most commercially relevant, while CNT- and graphene-based systems are better positioned for high-sensitivity near-clinical applications where manufacturing complexity is acceptable.

### 3.2. Metal-Oxide Nanomaterials

Metal-oxide nanomaterials have been extensively investigated as functional components in printed biosensors because of their intrinsic electrocatalytic activity, chemical stability, and compatibility with scalable printing processes. In particular, transition metal oxides such as CuO, ZnO, NiO, and TiO_2_ offer diverse redox properties and surface chemistries that enable both enzymatic and non-enzymatic sensing strategies on printed electrode platforms. Their performance in printed biosensors is strongly influenced by the particle size, morphology, composite design, and the choice of printing and post-processing methods.

Among metal oxides, CuO nanoparticles have emerged as one of the most widely used materials for non-enzymatic glucose sensing in printed electrochemical biosensors. CuO exhibits strong electrocatalytic activity toward glucose oxidation in alkaline media, coupled with good chemical robustness under repeated electrochemical cycling. High-performance CuO-based printed sensors have been demonstrated using inkjet-printable CuO nanoparticle inks deposited on metal or printed electrodes, achieving high sensitivities (2762.5 μA·mM^−1^·cm^−2^), wide linear ranges (0.05–18.45 mM), and low detection limits (~0.5 μM) while suppressing interference from common electroactive species through porous or nanostructured CuO films [140]. Cost-effective direct-write alternatives, such as micro-plotter printing, have also been shown to enable the deposition of CuO inks on flexible polymer substrates for disposable sensor fabrication without compromising electroanalytical performance with a sensitivity of 2419.8 μA·cm^−2^·mM^−1^ and linear range of 0.1–6.5 mM [141]. In addition, the integration of inkjet-printed CuO nanoparticles with an extended-gate field-effect transistor has demonstrated signal amplification effects, resulting in dual linear response regimes and extended dynamic ranges compared with conventional amperometric sensors [142].

To further enhance charge transport and catalytic efficiency, CuO has frequently been integrated into composite systems with carbon-based nanomaterials and noble metal catalysts. The incorporation of rGO or graphene provides continuous conductive pathways that reduce interfacial charge transfer resistance (Rct) and increase the electroactive surface area accessible to the analyte, whereas noble metals such as Pt or Pd anchored on the carbon scaffold introduce additional electrocatalytically active sites that lower the overpotential for glucose oxidation by facilitating the Cu^2+^/Cu^3+^ redox transition at reduced applied potential. Critically, the carbon scaffold also physically separates CuO nanoparticles during electrode fabrication and repeated electrochemical cycling, suppressing agglomeration that would otherwise reduce the accessible catalytic surface area over time—thus directly improving long-term operational stability. As a result, these synergistic nanocomposites consistently exhibit higher sensitivities, lower detection limits, and reduced operating potentials compared with CuO-only systems, with Pd-CuO/rGO and Pt-CuO/rGO composites achieving detection limits as low as 30 nM and 0.01 µM, respectively, on screen-printed electrodes [143,144,145]. The corresponding sensor performance metrics are summarized in Table 4, highlighting the effectiveness of composite engineering in overcoming the intrinsic conductivity limitations of metal oxide–based sensing materials. The Printing Method column in Table 4 illustrates the process constraints specific to each metal oxide: CuO nanoparticle systems are deposited predominantly by inkjet and screen printing, reflecting their compatibility with colloidal and paste-form inks; ZnO nanorods require post-printing hydrothermal growth combined with R2R or sputtered seed layers, because the nanoscale morphology critical to their sensing performance cannot be achieved by direct ink deposition alone; and TiO_2_ films require laser post-treatment after screen printing to develop the controlled porosity needed for functional sensing. This diversity of process routes within a single material class underscores that material selection and printing process must be co-optimized from the outset.

In addition, ZnO nanostructures have been widely explored as metal-oxide platforms for printed biosensors, owing to their high surface-to-volume ratio, facile enzyme functionalization, and favorable biocompatibility. The isoelectric point of ZnO (~9.5) allows electrostatic adsorption of negatively charged enzymes (e.g., GOx, isoelectric point ~4.2) at physiological pH, providing a simple yet effective immobilization route without chemical modification; additionally, the hydroxyl-rich ZnO surface supports silane-based covalent coupling via APTES, enabling more stable enzyme attachment for applications requiring extended operational lifetime. Printed biosensor platforms have combined the R2R printing of electrodes with the post-printing hydrothermal growth of ZnO nanowires or nanorods, enabling high-throughput manufacturing while maintaining nanoscale control over the sensing interface [146]. ZnO-based printed sensors have also been integrated into paper-based and microfluidic platforms to achieve clinically relevant glucose detection without the need for light-sensitive mediators, thereby improving operational stability [147]. Hybrid ZnO–CuO nanocomposites exploit the complementary properties of the two oxides: ZnO nanorods provide a high-surface-area scaffold with favorable enzyme-loading capacity and biocompatibility, while CuO nanoparticles contribute strong non-enzymatic electrocatalytic activity toward glucose oxidation in alkaline media through Cu^2+^/Cu^3+^ redox cycling. This synergistic combination yields sensing platforms with a sensitivity of 348.68 μA·mM^−1^·cm^−2^, a low detection limit of 0.40 μM, a response time below 5 s, and a linear range of 0.5 μM to 6.6 mM—a range that encompasses the clinically relevant fasting blood glucose threshold of 3.9–7.0 mM. Analytical performance was further validated by comparing sensor responses against a commercial clinical glucose analyzer using human serum samples, confirming acceptable agreement and supporting the practical translational potential of this composite architecture [148]. Moreover, other metal-oxide nanomaterials, such as NiO and TiO_2_, have also been explored as diverse sensing targets on printed platforms. In particular, NiO nanoparticles have demonstrated tunable electrochemical behavior that is strongly dependent on synthesis route, calcination temperature, and dispersion conditions, highlighting the critical role of material processing in governing electroactive surface area and electron transfer kinetics on screen-printed electrodes [149]. Printed TiO_2_ nanoparticle films, particularly when combined with post-printing laser treatments, enable controlled tuning of porosity and surface chemistry, thereby resulting in flexible sensors that ensure stable performance under mechanical deformation [150]. Notably, multicomponent oxide–carbon systems have been explored for simultaneous or multiplexed detection of clinically relevant analytes. In Pt/rGO/poly(3-aminobenzoic acid)-modified screen printed electrode, rGO serves as a conductive scaffold, Pt provides the catalytic activity, and the polymer layer enhances film stability and interfacial functionality, collectively enabling the sensitive electrochemical sensing of glucose and cholesterol [151].

Overall, metal-oxide nanomaterials provide a versatile and scalable material platform for printed biosensor fabrication, supporting both enzymatic and non-enzymatic detection schemes. A key practical distinction within this material class is the sensing strategy they enable: CuO and NiO are best suited to non-enzymatic configurations that eliminate the need for biorecognition element immobilization and offer inherently simpler fabrication and superior long-term stability under alkaline operating conditions, whereas ZnO and TiO_2_—with their biocompatible, hydroxyl-rich surfaces—are more appropriate for enzymatic and antibody-based sensing where surface functionalization chemistry is well-established. The principal limitations of metal oxide-based printed biosensors include their requirement for alkaline detection media in non-enzymatic configurations (limiting compatibility with physiological samples near neutral pH), their sensitivity to calcination temperature and particle morphology during ink preparation, and the additional post-deposition processing steps needed to develop functional nanostructures such as nanorods or nanowires. Continued advances in nanocomposite design, printing-compatible ink formulation, and post-deposition processing are expected to further enhance device performance while facilitating translation toward practical point-of-care diagnostic applications.

### 3.3. Metal Nanoparticles

Metal nanoparticles, particularly gold (AuNPs) and silver (AgNPs) nanoparticles, constitute an important class of functional materials for printed biosensors because of their high electrical conductivity, tunable surface chemistry, and strong electrocatalytic activity. Their nanoscale dimensions provide large electroactive surface areas, and their compatibility with inkjet printing, screen printing, and electrodeposition on printed electrodes facilitates scalable fabrication of high-performance sensing platforms.

Among metal nanoparticles, AuNPs have been the most extensively explored for printed biosensor applications, owing to their excellent biocompatibility, chemical stability, and ease of surface functionalization. In printed glucose sensing platforms, AuNPs promote rapid electron transfer and enable low-overpotential operation under both enzymatic and non-enzymatic detection schemes. Inkjet-printed AuNP inks on low-cost substrates such as paper have demonstrated that conductive sensing interfaces can be achieved under mild post-printing sintering conditions, enabling wide linear detection ranges (0.05–35 mM) and a lower detection limit (10 μM) [152]. Notably, AuNPs, which serve as signal amplification elements, have also been integrated with molecularly imprinted polymers (MIPs) for glucose selectivity and with polymeric interlayers for enhanced interfacial stability. These AuNP–MIP hybrid architectures have demonstrated ultrasensitive detection down to the nanomolar level, long-term operational stability over several weeks, and negligible cross-reactivity against common interfering species, highlighting their suitability for selective and durable printed biosensor platforms [153]. In enzymatic configurations, AuNP-modified printed electrodes integrated with Prussian blue and polymeric interlayers such as polytyramine enable efficient electron mediation and stable enzyme immobilization, resulting in sensitive glucose detection at low operating potentials suitable for flow-injection analysis. These hybrid electrode architectures achieve enhanced amperometric sensitivity (Table 4), reduced operating potentials (approximately −0.10 V), and sustained operational stability under continuous flow conditions, underscoring the effectiveness of combining AuNPs with redox-active and polymeric materials in printed biosensor design [154,155].

In addition to glucose sensing, AuNP-modified printed electrodes have been widely used as sensing platforms to detect clinical biomarkers and pathogens. AuNPs provide favorable microenvironments for antibody immobilization by offering a high surface area and versatile surface chemistry while also facilitating efficient electron mediation in enzyme-linked detection schemes. The well-established thiol–gold (Au–S) chemistry enables the self-assembled monolayer (SAM) formation of thiol-functionalized biomolecules—including thiolated aptamers, antibody fragments (Fab’), and cysteine-terminated peptides—directly on AuNP surfaces with high surface density and controlled orientation, a key advantage over non-oriented physical adsorption strategies that can reduce the binding affinity of antibodies by up to 80%. Consequently, AuNP-based printed immunosensors have enabled ultrasensitive detection of clinically relevant biomarkers such as B-type natriuretic peptide and prostate-specific antigen, achieving picogram-per-milliliter detection limits over wide linear ranges in sandwich-type and competitive immunoassays, as shown in Table 4 [156,157]. Notably, AuNP-based printed immunosensors have demonstrated robust analytical performance even when fabricated on flexible or recycled substrates, underscoring their suitability for low-cost, disposable point-of-care diagnostics. For example, the integration of AuNPs with CB on recycled PET-based screen-printed electrodes was shown to enable the ultrasensitive detection of SARS-CoV-2, achieving a detection limit of 46.2 fg·mL^−1^ at a per-unit production cost of only 0.29 USD [123]. Moreover, The integration of rGO/AuNP conjugates as electrochemical labels with immunomagnetic bead-based pre-concentration on screen-printed carbon electrodes enables reliable detection over a concentration range of 10^2^–10^6^ CFU·mL^−1^ with a detection limit of 89 CFU·mL^−1^ [158]. To expand the scope of analytes and improve their mechanical robustness, AuNPs have frequently been combined with carbonaceous nanomaterials, including graphene and carbon nanotubes. In these hybrid architectures, carbon materials act as conductive backbones and mechanically resilient supports, whereas AuNPs provide localized catalytic activity and biofunctionalization sites. Such synergistic composites enable the development of flexible printed biosensors with wide dynamic ranges and clinically validated performance, even under repeated bending or handling, as summarized in Table 4 [159,160,161].

AgNPs have emerged as cost-effective alternatives to AuNPs, offering strong electrocatalytic activity toward glucose oxidation and hydrogen peroxide reduction and conductivities on the order of 10^5^ S·m^−1^ when fabricated via in situ reduction or controlled electrodeposition on paper, textile, and polymer substrates [162,163,164]. Their principal advantage over AuNPs lies not in raw analytical performance, but in cost and scalability: AgNP inks are significantly cheaper to formulate and are compatible with high-throughput inkjet and screen printing processes, making them well-suited for disposable diagnostic applications where per-unit cost is a primary constraint. For biorecognition layer integration, the affinity of silver surfaces for thiol- and nitrogen-containing ligands enables stable aptamer and antibody immobilization without the need for SAM chemistry, supporting aptasensors and immunosensors with detection limits from the picomolar level to sub-100 CFU·mL^−1^ for targets including antibiotics, mycotoxins, and pathogenic bacteria [164,165,166]. AgNPs have also been exploited as electrochemical signal tags, where controlled anodic stripping of silver generates amplified, low-variance analytical signals—a strategy particularly effective for toxin and viral antigen detection, where signal amplification is more limiting than biorecognition layer selectivity [167]. Compared with AuNPs, AgNPs are more susceptible to oxidative degradation under ambient conditions, which constrains their shelf-life and motivates the use of backfilling and encapsulation strategies in long-term applications.

Overall, metal nanoparticle-based printed biosensors illustrate how nanoscale material properties can be effectively leveraged within scalable printing platforms. As summarized in the Printing Method column of Table 4, AuNP-based sensors are predominantly fabricated by screen printing for immunosensing applications requiring thick, high-conductivity electrode layers, with inkjet printing employed for precision deposition of AuNP inks on paper substrates; AgNP-based sensors similarly distribute across screen printing for high-throughput electrode fabrication and inkjet printing for aptamer-functionalized paper-based biosensors—a pattern that reflects the matching of each nanoparticle ink viscosity and particle size to the constraints of the target printing platform established in Table 1. AuNPs represent the gold standard for immobilization chemistry in printed biosensors—supporting thiol–Au SAM formation, EDC/NHS covalent coupling, and protein A/G-mediated antibody orientation—and are best suited to high-sensitivity clinical biosensing applications where selectivity and long-term operational stability are paramount. AgNPs offer a cost-effective alternative with adequate thiol-affinity for aptamer and antibody immobilization in short-term diagnostic formats, making them economically attractive for disposable, high-volume applications. The primary limitation of both platforms is susceptibility to surface oxidation and aggregation during ink formulation and storage, which constrains shelf-life and batch-to-batch reproducibility—challenges that must be addressed through controlled synthesis, surface passivation, and standardized storage conditions before broad clinical deployment becomes feasible. Continued advances in nanoparticle synthesis, surface modification, and hybrid material integration are expected to broaden the applications of printed biosensors toward practical point-of-care and field-deployable diagnostics.

### 3.4. Conductive Polymers for Printed Biosensor Platforms

Conductive polymers constitute an important class of functional materials for printed biosensors, offering intrinsic electrical conductivity, mechanical flexibility, and chemical tunability, characteristics that are difficult to achieve using inorganic materials alone. Among them, PEDOT:PSS has emerged as the most widely used polymeric ink owing to its excellent water processability, transparency, and compatibility with both inkjet and screen printing techniques. Recent research has predominantly focused on optimizing the chemical composition of PEDOT:PSS inks to simultaneously address their printing requirements and bioelectronic performance.

A key strategy for tuning the microstructure of PEDOT:PSS inks involves the use of secondary dopants and solvent additives. For example, Campos et al. reported viscosity-engineered PEDOT:PSS formulations compatible with screen printing on flexible substrates that enable rapid and stable electrochemical responses [168]. Lo et al. demonstrated inkjet-printed PEDOT:PSS electrodes, in which the combined use of ethylene glycol and surfactant additives improved both the electrical conductivity and film uniformity while maintaining stable resistance under repeated mechanical deformation [169]. Guo et al. further showed that multi-solvent doping strategies can induce pronounced PEDOT–PSS phase separation, yielding an ultra-low sheet resistance without compromising inkjet printability [170]. Furthermore, PEDOT:PSS has frequently been incorporated into nanocomposite inks to enhance both structural integrity and sensing functionality. Its polyelectrolytic characteristics allow it to function as a dispersant and stabilizing matrix for carbon nanomaterials, preventing aggregation and facilitating the formation of porous, electrically percolated networks. For example, graphene–PEDOT:PSS composites have been exploited for applications including electrochemical biosensing and temperature and strain monitoring, where the synergistic interaction between the two components produces properties unachievable by either alone: the conducting polymer matrix provides mechanical compliance and mixed ionic–electronic conductivity that maintains signal transduction under deformation, while the graphene phase provides a rigid, high-conductivity percolation network that prevents the conductivity drop typically observed in PEDOT:PSS films under repeated mechanical strain. The result is a composite ink that simultaneously satisfies the printability requirements of inkjet and screen printing platforms, the mechanical durability requirements of wearable biosensors, and the electrochemical performance requirements of sensitive analyte detection [171,172]. More recently, environmentally sustainable formulations based on non-hazardous solvents and additives such as dimethyl sulfoxide (DMSO) and isopropanol have been explored to address sustainability concerns in printed bioelectronics without compromising electrical performance or process compatibility [173]. In addition, PEDOT:PSS has served as an effective host matrix for enzymatic sensing layers. The mild aqueous processing conditions and mixed ionic–electronic conductivity of the polymer enable the direct incorporation or overprinting of enzyme systems while preserving biological activity. This entrapment-based immobilization strategy offers a key practical advantage: it requires no chemical modification of the biomolecule or the electrode surface, enabling single-step co-printing of the electrode and sensing layer. However, the absence of covalent bonding between the entrapped enzyme and the polymer matrix can lead to gradual biomolecule leaching during prolonged aqueous operation, limiting long-term stability compared with covalently coupled systems [174]. For applications such as printing on textiles, the ink should overcome the conditions of the substrate. Sinha et al. added DMSO and Triton X-100 as secondary dopants to induce spreading, a continuous network, and phase segregation on the ink to form a continuous network structure over curved, porous fibers and prevent unpredicted wicking [175].

In addition to PEDOT:PSS, conducting polymers such as PANI and polypyrrole (PPy) have been widely employed in printed biosensor platforms, owing to their intrinsic redox activities and responsiveness to environmental changes. PANI-based inks have been extensively investigated for pH and electrochemical sensing, with recent efforts emphasizing nanostructured morphologies and water-based or additive-free formulations to improve conductivity and printing robustness [176,177]. The amine-rich surface of PANI provides reactive sites for glutaraldehyde-mediated crosslinking of enzymes and antibodies, enabling covalent immobilization directly on the printed polymer film without additional surface modification steps—an advantage that distinguishes PANI from PEDOT:PSS, which relies primarily on entrapment and is less amenable to stable covalent bioconjugation. The incorporation of carbon nanomaterials into PANI matrices has further enhanced the charge transport and rate capability, underscoring the importance of hierarchical composite design in polymer-based sensors [178].

Although pristine PPy exhibits limited ink processability, recent advances in nanoparticle dispersion and surface modification strategies have enabled its effective use in printed biosensor platforms. Weng et al. demonstrated a fully inkjet-printed electrochemical platform by directly blending enzymes into stable PPy nanoparticle dispersions, enabling simultaneous printability and biofunctional integration [179]. Beyond simple entrapment, PPy electropolymerized directly on printed carbon or metal electrodes enables one-step co-immobilization of biomolecules during film growth, where the applied potential and monomer concentration govern polymer film thickness to nanometer precision—a critical parameter for minimizing the diffusion barrier between the analyte and the entrapped biomolecule while maintaining adequate mechanical stability of the immobilization layer. Building on these advances, hybrid polymer configurations and post-printing treatments have further expanded the functional design space for printed biosensors. For instance, Vacca et al. fabricated hybrid electrodes by inkjet printing a PEDOT:PSS layer followed by the electropolymerization of PANI, thereby integrating the high conductivity of PEDOT:PSS with the redox activity of PANI [180]. Similarly, Morais et al. demonstrated that the printing number of PANI and post-printing HCl vapor treatment of printed PANI/PEDOT:PSS bilayers effectively converted PANI into its conductive emeraldine salt form, leading to an enhanced humidity-sensing performance on paper substrates [181].

Conductive polymers such as PEDOT:PSS, PANI, and PPy provide versatile material platforms for printed biosensors, where compositional engineering, nanocomposite design, and post-printing treatments collectively facilitate the integration of printability, electrochemical functionality, and mechanical compliance. From an immobilization perspective, conductive polymers offer a distinctive advantage over inorganic nanomaterials: biorecognition elements can be directly entrapped within the polymer matrix during electropolymerization or co-printing, enabling simplified fabrication without additional surface modification. PEDOT:PSS supports enzyme entrapment via direct blending under mild aqueous conditions; PPy enables co-entrapment with nanometer-scale film thickness control during electropolymerization; and PANI provides amine-rich surfaces amenable to glutaraldehyde-mediated covalent crosslinking. However, compared with covalent coupling on metal or carbon surfaces, polymer entrapment typically yields lower long-term stability due to gradual biomolecule leaching. The principal practical limitation of conductive polymer-based biosensors is the sensitivity of their electrical properties to environmental conditions—particularly pH, ionic strength, and humidity—which complicates calibration and limits performance reproducibility in complex real-world sample matrices. Among the three platforms, PEDOT:PSS offers the best balance of printability and electrochemical stability for commercial-scale fabrication, while PANI and PPy are better suited to specialized applications requiring intrinsic redox activity or electropolymerization-based biorecognition layer integration. The representative performance metrics of conducting polymer-printed biosensors are summarized in Table 5, highlighting how material selection, processing strategies, and device architectures collectively influence analytical performance. The Printing Method column in Table 5 reflects the distinct processability characteristics of each polymer: PEDOT:PSS appears across both inkjet and screen printing configurations, consistent with its tunable viscosity range that accommodates both platforms; PANI is predominantly deposited by inkjet printing, reflecting its relatively low viscosity in dispersed form; and PPy is most commonly integrated via inkjet printing of nanoparticle dispersions or electropolymerization on pre-printed substrates, bypassing the direct printability limitations of pristine PPy. Collectively, Table 5 confirms that conductive polymer-based biosensors are most frequently fabricated by inkjet printing, in contrast to the screen printing dominance seen in carbon- and metal nanoparticle-based systems.

### 3.5. Dielectric Materials for Printed Biosensor Platforms

Recent advances in printable dielectric materials have focused on balancing high permittivity, process stability, and compatibility with low-temperature printing processes. A common strategy involves the incorporation of functional ceramic nanoparticles into polymer matrices, which enables tunable dielectric properties while maintaining ink processability and mechanical flexibility.

High-permittivity dielectric inks require improved dispersion uniformity to achieve optimal dielectric performance. Ca_2_Nb_3_O_10_ nanosheets dispersed in nanocomposite inks have been shown to enable reliable device operation when processed by aerosol jet printing, highlighting the compatibility of such systems with high-resolution printing techniques [182]. High-k dielectric requirements have been addressed using ferroelectric ceramic–polymer composites. Screen-printable BaTiO_3_- and Barium strontium titanate-based inks formulated with tailored polymer binders or sinterless solvent systems have enabled inkjet, screen, and aerosol jet printing of dielectric layers with low loss at radio and microwave frequencies, while avoiding post-printing sintering steps that are incompatible with flexible substrates [183,184,185]. In addition to nanoparticle-based composites, alternative chemical strategies and polymer-based dielectrics have enabled greater flexibility in controlling properties and fabricating printed dielectric layers and components. Sol–gel-derived oxide dielectrics offer a viable route for low-temperature processing while ensuring enhanced thermal and chemical stability on flexible substrates. In this regard, sol–gel-derived bismuth silicate inks have been shown to exhibit stable dielectric behavior when processed from simple precursor solutions, making them suitable for printed electronics that require thermal robustness without high-temperature annealing [186]. In contrast, polymer-based dielectric inks are designed to ensure process compatibility and device-level stability. Materials such as poly(methylsilsesquioxane), photoresist SU-8, poly(4-vinylphenol), and photoresist-based formulations enable facile patterning through inkjet and aerosol jet printing while supporting reliable operation in printed thin-film transistors and multilayer capacitor structures. These polymer dielectrics have also been shown to improve electrical stability and quality factors in fully additive fabrication schemes [187,188,189]. Recently, printed dielectric research has increasingly emphasized two-dimensional materials and sustainability as complementary design directions. For example, Worsley et al. demonstrated all-two-dimensional material capacitors based on water-based, biocompatible graphene and hexagonal boron nitride, achieving a high breakdown strength of 1.9 ± 0.3 MV/cm [190]. Notably, process scalability has emerged as a critical consideration for dielectric layers in printed electronics. R2R gravure printing of BaTiO_3_-based dielectric inks has demonstrated reliable integration into large-area circuits, highlighting the importance of ink rheology and solvent-evaporation control to suppress defects such as coffee-ring formation during high-speed printing [191,192]. These studies emphasize that the dielectric performance at scale is governed not only by material selection but also by ink formulation and drying kinetics.

Overall, recent advances in printable dielectric materials have demonstrated that the integration of ceramic nanoparticles, polymer matrices, two-dimensional materials, and scalable processing strategies enables dielectric layers that simultaneously satisfy the performance, process compatibility, and manufacturing requirements of printed biosensors and electronic platforms. The key advantage of this material class lies in its enabling role: dielectric layers do not contribute directly to sensing, but govern the electrical isolation, capacitive behavior, and mechanical integrity of multilayer biosensor architectures. The principal challenges for printable dielectrics are achieving sufficiently high permittivity and low leakage current without high-temperature sintering that is incompatible with flexible substrates, and suppressing defect formation (coffee-ring effect, pinholes) during high-speed R2R deposition. Polymer-based dielectrics currently offer the best compatibility with low-temperature flexible substrate processing, while ceramic–polymer nanocomposites provide higher permittivity at the cost of increased ink formulation complexity. As printed biosensors advance toward fully integrated architectures incorporating on-board electronics and signal conditioning, the performance and reproducibility of the dielectric layer will become an increasingly critical determinant of overall device functionality.

**Figure 4:**
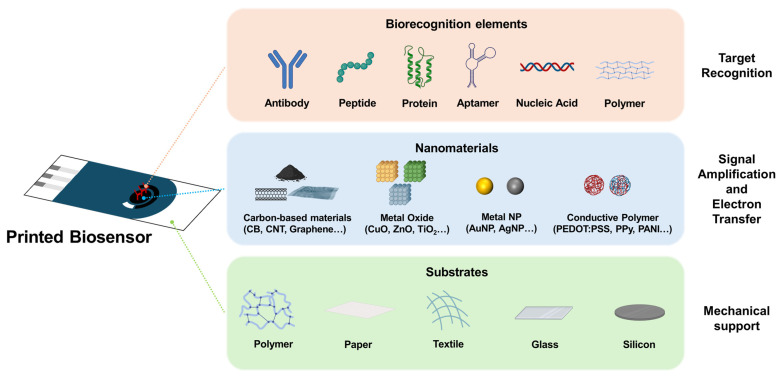
Component architecture and functional roles of nanomaterials within printed electrochemical biosensors. The schematic illustrates the three principal layers comprising a printed biosensor and their respective functions within the integrated device. (**Bottom**) The substrate layer provides mechanical support and defines the physical form factor of the device; substrates employed across the printed biosensors reviewed include polymeric films (PET, PI, PDMS), paper, textile, glass, and silicon, each imposing distinct requirements on printing compatibility, flexibility, and post-processing conditions. (**Middle**) The nanomaterial functional layer encompasses the four material classes central to this review—carbon-based nanomaterials (CB, CNT, graphene derivatives), metal oxides (CuO, ZnO, TiO_2_), metal nanoparticles (AuNP, AgNP), and conductive polymers (PEDOT:PSS, PPy, PANI)—which collectively serve as the signal amplification and electron transfer interface between the printed electrode and the biorecognition layer, while simultaneously providing the surface chemistry required for stable biomolecule immobilization. (**Top**) The biorecognition element layer confers target selectivity through specific molecular recognition; elements employed across the reviewed platforms include antibodies, peptides, proteins, aptamers, nucleic acids, and polymers. The dashed connectors linking the printed biosensor device (**left**) to each layer emphasize that these components are not independent material choices but are co-integrated within a single fabricated architecture whose analytical performance is determined by their collective design. The figure was prepared using BioRender.

Across the nanomaterial classes reviewed in Section 3.1, Section 3.2, Section 3.3, Section 3.4 and Section 3.5, a unifying principle emerges: the analytical performance of a printed biosensor is not determined by any single material choice, but by the co-integrated design of the substrate, the nanomaterial functional layer, and the biorecognition element. Figure 4 summarizes this device-level perspective. The substrate defines the mechanical and processing constraints of the platform; the nanomaterial layer amplifies the electrochemical signal, enhances the electroactive surface area, accelerates electron transfer kinetics, and provides the surface chemistry that governs immobilization; and the biorecognition element confers the molecular selectivity required for target detection. Understanding how these three layers interact—and how each nanomaterial class fulfills its role within this architecture—is essential for translating the material advances described in this section into high-performance, manufacturable biosensor platforms.

## 4. Challenges and Future Perspectives

Despite substantial progress, several issues limit the broader translation of printed biosensors from laboratory demonstrations to practical diagnostic platforms. The first challenge is the strong interdependence between the printing process and material behavior. Ink rheology, particle dispersion, surface tension, solvent evaporation, and substrate wettability must be carefully matched to each printing method, and small deviations often lead to defects such as nozzle clogging, coffee-ring formation, overspreading, line distortion, or poor interlayer registration. These problems have become more pronounced in multilayer and hybrid devices, where electrical, catalytic, and biological components must be integrated without damaging the previously deposited layers. In parallel, long-term ink stability remains a persistent concern, particularly for formulations containing nanomaterials, enzymes, antibodies, aptamers, and mediators. Aggregation, viscosity drift, phase separation, and biomolecular degradation can reduce both printability and analytical reproducibility. Post-printing treatment presents an additional bottleneck because conductivity or catalytic activity often requires curing, sintering, reduction, or chemical activation, whereas biological functionality generally favors mild processing conditions.

The second challenge concerns sensor reliability under realistic operating conditions. Although many printed biosensors report impressively low detection limits, a critically low LOD does not automatically translate into practical diagnostic utility. For a given target analyte, the clinically or physiologically relevant concentration range must be the primary benchmark against which sensor performance is evaluated. For example, glucose monitoring in blood requires a working range of approximately 2–30 mM, rendering sub-micromolar LODs analytically irrelevant for this application, even if they represent a technical achievement in terms of electrode design. Similarly, cortisol in sweat is present at concentrations of 8–140 nM, and cardiac troponin I in serum is clinically significant at the pg·mL^−1^ level; a biosensor optimized for femtomolar detection of these analytes in buffer may still fail to perform reliably in the presence of the complex interferent matrix of real biological samples. Furthermore, performance reported in idealized laboratory conditions—typically using phosphate-buffered saline spiked with a single analyte—frequently does not translate to equivalent performance in whole blood, sweat, saliva, or urine, where protein fouling, ionic strength variation, and competing redox-active species substantially degrade both sensitivity and selectivity. Accordingly, future work should place greater emphasis on reproducibility across fabrication batches, storage stability under ambient conditions, and systematic validation in clinically relevant sample matrices, rather than prioritizing record-low detection limits as the primary figure of merit. Standardized reporting of linear range, recovery rate in spiked real samples, and inter-electrode reproducibility (expressed as relative standard deviation) would substantially improve the comparability of results across the field and better reflect the practical readiness of printed biosensor platforms for point-of-care deployment.

Biocompatibility and regulatory acceptance must also be carefully considered, particularly for devices intended for skin contact, continuous monitoring, and use with biological fluids. Residual solvents, nanomaterial leaching, and degradation byproducts may all affect safety and must be systematically evaluated as part of a regulatory submission. Beyond safety, the pathway to clinical deployment imposes several additional requirements that are rarely discussed in the printed biosensor literature. Shelf-life stability is a fundamental prerequisite: printed biosensors incorporating enzymes, antibodies, or aptamers must demonstrate retained analytical performance over storage periods of at least 6–12 months under defined temperature and humidity conditions, a requirement that is rarely reported in academic studies and that remains a major gap between laboratory demonstrations and market-ready devices. Batch-to-batch reproducibility must meet the tight tolerances demanded by clinical quality standards—typically a coefficient of variation below 5–10% across independently fabricated lots—which necessitates rigorous control of ink formulation, printing parameters, and post-processing conditions throughout the manufacturing workflow. Calibration and validation protocols represent another underaddressed requirement: unlike laboratory instruments that can be recalibrated on demand, disposable printed biosensors must either incorporate on-device calibration features or demonstrate sufficiently low lot-to-lot variation to enable factory calibration, both of which impose significant constraints on device design and manufacturing precision. Assay complexity is also a practical concern, as multi-step protocols involving sequential reagent addition, incubation, and washing steps are generally incompatible with point-of-care deployment; printed biosensor designs must therefore prioritize minimal user intervention and ideally achieve single-step or reagent-free operation. Finally, regulatory expectations for in vitro diagnostic devices—including ISO 13485 [193] quality management, analytical validation according to CLSI guidelines, and device classification under FDA 510(k) or EU IVDR frameworks—demand levels of documentation, traceability, and clinical evidence that go well beyond what is typically reported in proof-of-concept studies. Commercialization of printed biosensors will therefore require not only continued advances in materials and fabrication but also a fundamental shift in how performance is reported, validated, and benchmarked against established clinical standards.

Reproducibility and standardization represent a distinct and underaddressed challenge in the printed biosensor field. Batch-to-batch consistency is governed by a complex interplay of factors that span ink formulation, printing process, substrate properties, and post-printing treatment. At the ink level, variability in nanoparticle size distribution, surface functionalization density, and dispersion stability between synthesis batches directly translates into inconsistent rheological behavior, which in turn affects droplet formation in inkjet printing, paste transfer in screen printing, and aerosol generation in aerosol jet printing. Even within a single print run, solvent evaporation from the ink reservoir can cause progressive viscosity drift, altering film thickness and electrode morphology over time. At the substrate level, lot-to-lot variation in surface energy, roughness, and porosity—particularly for paper and textile substrates—introduces additional variability in ink spreading, adhesion, and drying kinetics. Post-printing processes such as thermal sintering, chemical reduction, or laser treatment are similarly sensitive to equipment calibration and ambient conditions, leading to run-to-run differences in conductivity and electroactive surface area. For biosensor-specific layers, the immobilization of enzymes, antibodies, or aptamers introduces further sources of variability, as biomolecule activity, surface coverage, and orientation are highly sensitive to temperature, pH, ionic strength, and the precise surface chemistry of the underlying electrode. Collectively, these factors make it difficult to achieve the tight performance tolerances required for clinical diagnostic applications, where inter-device coefficient of variation is typically required to be below 5–10%. Addressing these barriers will require the development of standardized ink certification protocols, tighter process control through in-line monitoring and closed-loop feedback systems, and the adoption of common benchmarking standards—including reference analytes, sample matrices, and reporting metrics—to enable meaningful cross-laboratory and cross-platform comparisons.

The translation of laboratory-scale printed biosensor prototypes into industrial production presents several practical challenges that extend beyond material and device optimization. Ink formulations optimized at the milliliter scale frequently exhibit altered rheological behavior when scaled to production volumes, requiring re-optimization of viscosity, particle dispersion, and solvent composition to maintain printability and film uniformity across large batches. Equipment transition from benchtop printers to industrial R2R or high-throughput screen-printing lines introduces additional variability through differences in shear rate, drying kinetics, web tension, and registration accuracy, all of which must be systematically characterized and controlled. Automated in-line quality control systems—including optical inspection, electrical probing, and electrochemical spot-testing—will be essential to identify and reject out-of-specification devices during continuous manufacturing runs without interrupting production flow. Furthermore, the cost structure of industrial fabrication demands that material consumption, energy use, and yield loss be rigorously optimized, particularly for biosensor designs incorporating expensive nanomaterials such as AuNPs or specialty conductive polymers, where material cost per device can become prohibitive at scale.

Looking ahead, this research field is advancing toward more integrated design strategies in which material selection, printing compatibility, and application requirements are considered simultaneously. Hybrid and composite inks may play an increasingly important role because they allow conductivity, catalytic activity, mechanical compliance, and biofunctionality to be distributed across multiple components rather than relying on a single material. At the same time, sustainable formulations based on water-processable systems, bio-derived polymers, and low-temperature processing are gaining importance as printed biosensors move closer to large-scale deployments. Future advances will likely be driven by platforms that combine multiplexed sensing, wearable or soft-device formats, and compatibility with portable or wireless readout systems. Despite progress in individual printed components, the realization of fully integrated printed devices that combine electrochemical sensing cells, on-board electronics, and microfluidic sample handling within a single fabricated unit remains a significant unmet challenge. At the electrochemical level, integrating working, reference, and counter electrodes with stable and drift-free electrochemical behavior onto a single flexible substrate requires careful management of cross-contamination between printed layers and long-term potential stability of printed pseudo-reference electrodes, which typically exhibit greater drift than conventional Ag/AgCl references. Incorporating printed electronic components—such as amplifiers, analog-to-digital converters, and wireless transmission modules—into the same substrate introduces additional constraints, as the sintering or curing temperatures required for conductive ink processing are often incompatible with pre-deposited biological or polymeric layers. At the microfluidic level, printed channel structures must reliably control sample flow, metering, and mixing without external pumps or valves, yet achieving consistent channel geometry and wettability across printing runs remains difficult due to ink spreading variability and substrate surface energy inhomogeneity. The interface between the microfluidic layer and the electrochemical sensing surface is particularly critical, as improper sealing or misalignment can introduce dead volumes, bubble entrapment, or inconsistent analyte delivery that directly compromises analytical performance. Furthermore, the multiplexing of several sensing elements within a single device—as required for the simultaneous detection of multiple biomarkers—demands precise spatial registration of different functional inks across multiple printing passes, a requirement that pushes the limits of current printing accuracy and interlayer compatibility. Addressing these integration challenges will require coordinated advances in multi-material printing strategies, low-temperature-compatible conductive and dielectric inks, self-aligned fabrication approaches, and standardized interconnect designs that enable reliable assembly of printed electrochemical, electronic, and fluidic subsystems into a single manufacturable platform. In this context, the most meaningful progress will be achieved not simply from improving individual performance metrics but through achieving a practical balance between sensitivity, stability, manufacturability, cost, and regulatory readiness.

## Figures and Tables

**Table 1:** Comparison of technical parameters for major printing technologies used in biosensor fabrication.

Parameter	Inkjet	Screen Printing	Aerosol Jet	Roll-to-Roll
Ink Viscosity (mPa·s)	1–40	1000–10,000	1–1000	50–500
Particle Size (nm)	<200	<10,000	<500	<5000
Substrate compatibility	Flexible (PET, paper, fabric)	Rigid and Flexible (ceramic, plastic, paper)	3D Conformal (non-planar surfaces)	Flexible only (PET, paper)
Surface Tension (mN/m)	25–50	Not critical	<30	25–50
Resolution (μm)	20–50	50–100	10–20	20–75
Thickness (μm)	0.1–2	5–25	0.1–5	0.5–12
Printing Speed	Medium	High	Low	Very High
Scalability/Throughput	Medium	High	Low	Very High
Biosensor Deployment Status	Lab-to-clinic prototypes	Commercial mass production	R&D/prototyping only	Pilot-scale (electrode only)

**Table 2:** Key advantages and limitations of major printing technologies used in biosensor fabrication.

Technology	Key Advantages	Key Limitations
Inkjet	Non-contact, digital patterning (maskless) Low material waste High resolution (20–50 μm) Rapid prototyping capability Multi-material printing in single pass Low-temperature processing	Nozzle clogging with high-viscosity inks Limited to particle size < 200 nm Thin film deposition only (0.1–2 μm) Coffee-ring effect during drying Slower than contact printing methods
Screen Printing	High throughput, low cost Thick film deposition (5–25 μm) Wide ink viscosity range Well-established industrial process Compatible with various substrates Excellent reproducibility	Limited resolution (50–100 μm) Requires physical mask/screen Not suitable for 3D/non-planar surfaces Screen wear and mesh clogging Design changes require new screens
Aerosol Jet	Highest resolution (10–20 μm) 3D conformal printing capability Large stand-off distance (2–5 mm) Wide ink viscosity tolerance Non-contact deposition Complex geometry printing	Low throughput High equipment cost Complex process optimization Limited scalability Primarily for R&D/prototyping Overspray issues
Roll-to-Roll	Highest throughput (up to 15 m/s) Continuous production (>150 m rolls) Lowest cost per unit at scale Excellent for mass production High reproducibility Compatible with flexible substrates	Requires dedicated gravure cylinders High initial tooling cost Not suitable for rigid substrates Design changes require new cylinders High shear forces may damage biomolecules Limited to relatively simple patterns

**Table 3:** Summary of carbon nanomaterial-based printed biosensors.

Category	Material	Printing Method	Substrate	Analyte	LOD	References
Carbon Black	CB/Prussian Blue	Screen printing	Polyester	H_2_O_2_	0.3 μM	[120]
	CB/Cu-MOF	Screen printing	Polyester	Nitrite	0.084 μM	[121]
	CB/PB/CNT/Chitosan	Screen printing	PET	Glucose	20 μM	[122]
	CB/AuNPs	Screen printing	PET	SARS-CoV-2	101 fg/mL	[123]
	CB/PdNPs	Screen printing	PET	Parkinson biomarkers	0.051 μM	[124]
CNT	Nafion/MWCNT	Inkjet printing	PET	Dopamine	0.1 μM	[126]
	SWCNT	R2R gravure	PET	Touch	N/A	[109]
	MWCNT/PPtNPs/GOx	Screen printing	SPE	Glucose	2.50 μM	[127]
	f-MWCNT/AgNPs	Screen printing	GCE	Dopamine	0.2778 μM	[128]
	MWCNT/Uricase	Screen printing	PET	Uric acid	0.33 μM	[129]
	AuNPs/MWCNT/Nafion	Screen printing	SPE	Dopamine	0.01 μM	[130]
Graphene	PEDOT:PSS/Graphene	Inkjet printing	Paper	Glucose	50 μM	[131]
	Graphene	Inkjet printing	PI	Glucose	100 nM	[132]
	rGO	Screen printing	SPE	Cortisol/Lactate	0.1 ng/mL/0.1 mM	[133]
	Graphene	Stencil printing	PET	Glucose	16.42 μA/mM/cm^2^	[14]
	LIG/PEDOT/AuNPs	Laser-induced graphene	PI	Glucose	2 μM	[134]
	LIG	Laser-induced graphene	PI	Glucose/Lactate	14.9 μM/2.4 mM	[135]
	LIG/Graphene-PEDOT:PSS	Screen printing	PI	Dopamine/IL-6	2.6234 pg/mL	[136]
	LIG	Laser-induced graphene	Paper	Glucose	25–50 μM	[137]
QDc	GQD/AuNPs	Screen printing	SPE	HIV RNA	1 fg/mL	[138]
	N-GQD	Screen printing	SPE	Histidine	0.1 nM	[139]

**Table 4:** Summary of metal nanomaterial-based printed biosensors.

Category	Material	Printing Method	Substrate	Analyte	LOD	References
Metal Oxide	CuO NPs	Inkjet	Metal/Printed Electrode	Glucose	~0.5 µM	[140]
	CuO NPs	Micro-plotter	PET	Glucose	N/A	[141]
	CuO + EG-FET	Inkjet	Polyimide (PI)	Glucose	0.01 mM	[142]
	Pd-CuO/rGO	Screen Printing	SPCE	Glucose	30 nM	[143]
	Pt-CuO/rGO	Screen Printing	SPCE	Glucose	0.01 µM	[144]
	CuO/graphene	Screen Printing	SPCE	Glucose	34.3 nM	[145]
	ZnO NR (Roll-to-roll + Hydrothermal)	Roll-to-roll	N/A	N/A	N/A	[146]
	ZnO (Paper/Microfluidic)	N/A	Paper	Glucose	N/A	[147]
	ZnO NR/CuO NPs	Hydrothermal (+Sputtering seed)	FTO	Glucose	0.40 µM	[148]
	NiO NPs	Screen Printing	SPCE	N/A	N/A	[149]
	TiO_2_ NPs	Screen printing + Laser irradiation post-treatment	PET (Polyethylene Terephthalate)	UV light/Ethanol (gas)	N/A	[150]
	Pt/rGO/poly	Screen Printing	SPCE	Glucose, Cholesterol	Glucose: 44.3 µM/Cholesterol: 40.5 µM	[151]
Gold (Au)	AuNP ink	Inkjet	Paper	Glucose	10 µM	[152]
	AuNP–MIP	N/A	N/A	Glucose	1.25 nM	[153]
	GOx/AuNPs/Pty/PB	Screen Printing	SPCE	Glucose	N/A	[154]
	PB	N/A	Au-SPE	H_2_O_2_	N/A	[155]
	AuNPs/Diazonium	Screen Printing	SPCE	BNP	4 pg/mL	[156]
	AuNPs/Chitosan	Screen Printing	SPE	PSA	0.001 ng/mL	[157]
	AuNP + IL	Screen Printing	SPCE	*S. pullorum*	89 CFU/mL	[158]
	AuNPs/Graphene	N/A	Graphene paper	*E. coli* O157:H7	N/A	[159]
	AuNPs + MWCNTs	Screen Printing	SPCE	HbA1c	N/A	[160]
	PB-CS-AuNP	N/A	N/A	Histamine	N/A	[161]
Silver (Ag)	AgNPs	Inkjet	Paper/Textile	Glucose, H_2_O_2_	N/A	[162]
	AgNPs/HMDA-PEG	Screen Printing	SPCE	H_2_O_2_	1.5 µM	[163]
	AgNP/Aptamer	Inkjet	Paper	Ampicillin	10 µg/mL	[164]
	AgNP/Aptamer	Screen Printing	SPCE	Ochratoxin A	N/A	[165]
	AgNP/Aptamer	Screen Printing	SPCE	*E. coli*	150 CFU/mL	[166]
	AgNP signal tag	N/A	N/A	Shiga toxin-1	N/A	[167]

**Table 5:** Summary of conducting polymer nanomaterial-based printed biosensors.

Category	Material	Printing Method	Substrate	Analyte	LOD	References
PEDOT:PSS	EG, DMSO, etc.	Screen printing	PET	H_2_O_2_	0.97 µM	[168]
	Ethylene glycol, Triton X-100	Inkjet printing	PDMS, TPU	ECG, pulse sensing (health monitoring)	N/A	[169]
	DMSO, ethanol, diethylene glycol	Inkjet printing, sponge stencil technique	Non-woven PET fabric	N/A (for wearable electronics)	N/A	[170]
	Graphene	Inkjet printing	SPCE	H_2_O_2_, NAD^+^/NADH, K_4_Fe(CN)_6_	194.37/17.28/0.36 μM	[171]
	Graphene	Inkjet printing	Polyurethane	Temperature	N/A (0.06% per degree Celsius sensitivity)	[172]
	DMSO, isopropanol	Inkjet printing	Non-woven fabric	touch	N/A	[173]
	Glucose oxidase, horseradish peroxidase	Piezoelectric inkjet printing	ITO-coated PET	Glucose	270 μM (signal to noise ratio 3)	[174]
	DMSO, Triton X-100	Screen printing	Finished textile	ECG	N/A	[175]
PANI	Acrylic dispersion	Inkjet printing	Screen-printed carbon ink layer onto Valox	pH	N/A	[176]
	DMSO, ionic liquids	Inkjet printing	Various	N/A	N/A	[177]
	C-MWCNT	Mask-assisted spray-coating	Various (PET, paper, wood)	N/A (microsupercapacitor)	N/A	[178]
PPy	HRP, GoD	Inkjet printing	Flexible substrate	H_2_O_2_	H_2_O_2_: 10 μM–10 mM Glucose: 1–5 mM	[179]
	PEDOT:PSS	Inkjet printing	PEN	pH	Below 8–9	[180]
	PEDOT:PSS	Inkjet printing	Paper	Humidity	N/A	[181]

## Data Availability

No new data were created or analyzed in this study.
